# Prophylactic closed‐incision negative‐pressure wound therapy is associated with decreased surgical site infection in high‐risk colorectal surgery laparotomy wounds

**DOI:** 10.1111/codi.14350

**Published:** 2018-08-20

**Authors:** T. Curran, D. Alvarez, J. Pastrana Del Valle, T. E. Cataldo, V. Poylin, D. Nagle

**Affiliations:** ^1^ Division of Colon and Rectal Surgery Department of Surgery Beth Israel Deaconess Medical Center Boston Massachusetts USA

**Keywords:** Closed incision negative pressure therapy, surgical site infection, quality improvement

## Abstract

**Aim:**

Surgical site infection in colorectal surgery is associated with significant healthcare costs, which may be reduced by using a closed‐incision negative‐pressure therapy device. The aim of this study was to assess the impact of closed‐incision negative‐pressure therapy on the incidence of surgical site infection.

**Method:**

In this retrospective cohort study we evaluated all patients who had undergone high‐risk open colorectal surgery at a single tertiary care centre from 2012 to 2016. We compared the incidence of surgical site infection between those receiving standard postoperative wound care between 2012 and 2014 and those receiving closed‐incision negative‐pressure therapy via a customizable device (Prevena Incision Management System, KCI, an Acelity company, San Antonio, Texas, USA) between 2014 and 2016. A validated surgical site infection risk score was used to create a 1:1 matched cohort subset.

**Results:**

Negative pressure therapy was used in 77 patients and compared with 238 controls. Negative pressure patients were more likely to have a stoma (92% *vs* 48%, *P* < 0.01) and to be smokers (33% *vs* 15%, *P* < 0.01). Surgical site infection was higher in control patients (15%, *n* = 35/238) compared with negative pressure patients (7%, *n* = 5/77) (*P* = 0.05). On regression analysis, negative pressure therapy was associated with decreased surgical site infection (OR 0.27; 95% CI 0.09–0.78). These differences persisted in the matched analysis.

**Conclusion:**

Negative pressure therapy was associated with decreased surgical site infection. Negative pressure therapy offers significant potential for quality improvement.

## What does this paper add to the literature?

The role of closed‐incision negative‐pressure therapy in reducing the risk of surgical site infection in colorectal surgery is unclear. This study suggests that this technology decreases the risk of infection in high‐risk patients undergoing open colorectal surgery.

## Introduction

Surgical site infection (SSI) in colon and rectal surgery is common and costly. A 2009 national registry study showed that 4% of patients undergoing colorectal procedures were diagnosed with SSI on their index admission 1. Using the National Surgical Quality Improvement Program (NSQIP) database, Kiran and colleagues demonstrated that this rate increased to 14% when patients were followed up for 30 days after surgery 2. SSI occurring during the index admission is associated with a near doubling of hospital length of stay and cost of admission 1. Readmissions following colorectal surgery are similarly expensive, with SSI noted as the second most common cause for hospital readmission after colectomy 3, 4.

Not surprisingly, interventions that might reduce the incidence of SSI have been extensively investigated. One such intervention is the use of closed‐incision negative‐pressure therapy (CINPT) dressings. Theoretically, these dressings prevent external contamination of the incision and also encourage tissue apposition, tissue perfusion and the removal of fluid and infectious material from the incision 5. A recent wound bed mRNA study has also shown that CINPT influences growth factors, inflammatory cytokines and matrix metalloproteinases to promote wound healing 6. Several reviews and meta‐analyses have shown promising results with CINPT 5, 7, 8. However, the majority of studies have been small with heterogeneous patient populations, making it difficult to draw definitive conclusions.

This study aimed to assess the effect of prophylactic CINPT on the incidence of SSI in a cohort of high‐risk patients undergoing open colorectal surgery. We hypothesized that this high‐risk group would demonstrate maximum benefit from such a technology.

## Method

### Study design/patient selection

We identified all NSQIP‐reviewed patients undergoing open abdominal colorectal surgery within our Division of Colon and Rectal Surgery between 2012 and 2016. The use of NSQIP‐reviewed patient records facilitated the application of standardized criteria for postoperative adverse events as well as complete capture of these events to 30 days postoperatively, including readmissions to outside institutions 9. Patients at high risk for SSI were then selected for study. Patients were classified as high risk if they had one or more of the following factors: pre‐ or postoperative stoma, diabetes mellitus, obesity, preoperative steroid or immunosuppressant use and/or a contaminated/dirty wound 10, 11. The risk of SSI was assessed using the validated SSI risk score devised by van Walraven and colleagues, which utilizes a combination of preoperative and operative parameters 12. Patients undergoing unplanned reoperation within 30 days of the index procedure were excluded from the analysis 13. This study was approved by the Institutional Review Board of the Beth Israel Deaconess Medical Center.

### Intervention group and historical control group

Commencing in May 2014, high‐risk patients undergoing open abdominal colorectal surgery received prophylactic CINPT using a customizable device (Prevena Incision Management System, KCI, an Acelity company, San Antonio, Texas, USA) at the discretion of the operating surgeon. The vacuum device was applied over the intact incision in the operating room under sterile conditions and left in place for 5–7 days. It was subsequently removed in hospital or in the outpatient setting as appropriate. Whilst in place, the vacuum device was set to provide suction at a pressure of −125 mm Hg 8. Traditional, reusable suction pump vacuum devices were initially used but single‐use devices which are more cost‐effective were subsequently routinely employed 14, 15. The patients receiving CINPT were compared with high‐risk patients undergoing similar procedures from January 2012 to June 2016. Patients in whom the wound was not closed were excluded.

All patients received the same perioperative care. We routinely use both mechanical and oral antibiotic bowel preparation for all elective colorectal resections 16. All patients receive intravenous antibiotics within an hour of surgical incision and these were discontinued within 24 h of surgery 17. Chlorhexidine–alcohol‐based scrub is used for site preparation, and a wound protector is utilized for specimen extraction 18, 19. Gloves were changed after completion of the anastomosis or any contaminated portion of the case.

### SSI risk‐matched subset analysis

In order to compare the study group with controls with similar SSI risk we performed a subset analysis whereby the control group was stratified according to the van Walraven SSI risk score and randomly matched 1:1 with the study group.

### Outcome measures

Our primary outcome measure was a composite of superficial SSI, deep SSI or dehiscence at 30 days as assigned by the NSQIP 20. Organ space SSIs were excluded from this composite measure as CINPT is not thought to influence this. Secondary outcomes included length of stay, unplanned readmission and organ space SSI.

### Statistical analysis

Patient demographics, comorbidities and perioperative details were extracted from the NSQIP database for analysis. The CINPT study group and control group were compared with respect to perioperative characteristics and postoperative outcomes. The chi‐square test or Fisher's exact test were used to compare categorical variables. The two‐tailed independent samples *t*‐test or Wilcoxon rank‐sum test was used to compare continuous variables. Stratification on the van Walraven SSI risk score was then used to identify 1:1 matched cohorts of patients receiving CINPT and those that did not.

Multivariable logistic regression was performed to determine independent predictors of SSI. Patients with SSI were compared with those who did not develop SSI. All variables with *P* < 0.10 on bivariate analysis were included in the model. These included CINPT usage (*P* = 0.05), dialysis dependence (*P* = 0.05) and operative time (*P* = 0.06). There were no missing data. Backward stepwise elimination was used to determine final independent predictors with variables eliminated for *P* > 0.05. Model discrimination was assessed using *c*‐statistics with a *c*‐statistic of 1.0 denoting perfect predictive power and a *c*‐statistic of 0.5 denoting a prediction equivalent to random chance. The Hosmer–Lemeshow test was used to assess model calibration 21. Throughout all analyses, statistical significance was determined by a *P*‐value of < 0.05. All analyses were conducted using IBM SPSS Statistics version 24.0.0.1 for Macintosh (IBM Corp., Armonk, New York, USA).

## Results

### Patient cohort

**Figure 1:**
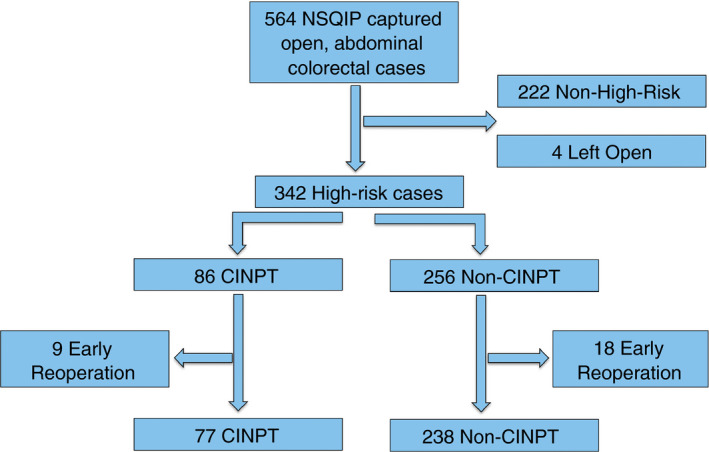
Patient selection.

The NSQIP database captured 564 open abdominal colorectal procedures over the study period. After exclusions, 315 patients were included for analysis; 77 receiving CINPT (24%) and 238 non‐CINPT (76%) (Fig. 1). From the time of introduction of CINPT, 46% (*n* = 86/188) of patients meeting the criteria received CINPT.

### Comparison of the CINPT group with the control group

**Figure 2:**
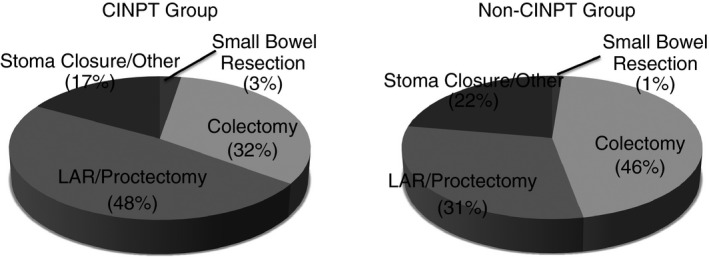
Surgical procedure: CINPT *vs* non‐CINPT.

A comparison of the demographic and preoperative details for the CINPT and control groups is shown in Table 1. The patients were similar with respect to preoperative characteristics, although the CINPT group had a larger proportion of active smokers (CINPT *vs* control, 33% *vs* 15%; *P* < 0.01). The surgical procedures performed for each group are shown in Fig. 2 and operative details are given in Table 2. The mean predicted SSI rate for the CINPT group was 20.0% (SD 5.7%) compared with 15.0% (SD 5.4%) for the control group (*P* < 0.01).

**Table 1:** Preoperative and demographic characteristics of CINPT *vs* non‐CINPT patients

	All *N* = 315	CINPT *N* = 77	Non‐CINPT *N* = 256	*P*‐value
Age (years), mean (SD)	57 (15)	56 (15)	58 (16)	0.36
Female gender, *n* (%)	140 (44)	31 (40)	109 (46)	0.43
BMI ≥ 30 kg m^–2^, *n* (%)	144 (46)	31 (40)	113 (48)	0.29
Steroid use, *n* (%)	104 (33)	23 (30)	81 (34)	0.58
Diabetes mellitus, *n* (%)	73 (23)	14 (18)	59 (25)	0.28
Race, *n* (%)				
White	262 (83)	66 (86)	196 (82)	0.71
Unknown	24 (8)	6 (8)	18 (8)	
Hawaiian/Islander	1 (0)	0 (0)	1 (0)	
Black	18 (6)	2 (3)	16 (7)	
Asian	6 (2)	2 (3)	4 (2)	
American Indian/Alaskan	4 (1)	1 (1)	3 (1)	
Smoker, *n* (%)	60 (19)	25 (33)	35 (15)	<0.01
Dependent functional status, *n* (%)	18 (6)	3 (4)	15 (6)	0.58
History of severe COPD, *n* (%)	15 (5)	4 (5)	11 (5)	0.77
Dialysis, *n* (%)	7 (2)	0 (0)	7 (3)	0.20
Disseminated cancer, *n* (%)	20 (6)	4 (5)	16 (7)	0.79
Open wound, *n* (%)	29 (9)	6 (8)	23 (10)	0.82
Weight loss, *n* (%)	40 (13)	10 (13)	30 (13)	1.00
Bleeding disorder, *n* (%)	23 (7)	3 (4)	20 (8)	0.22
Preoperative transfusion, *n* (%)	9 (3)	2 (3)	7 (3)	1.00
Any preoperative SIRS/sepsis, *n* (%)	31 (10)	11 (15)	20 (9)	0.18
ASA class, *n* (%)				
ASA 1	1 (0)	0 (0)	1 (0)	0.65
ASA 2	106 (34)	24 (31)	82 (35)	
ASA 3	180 (57)	48 (62)	132 (56)	
ASA 4	28 (9)	5 (7)	23 (10)	

BMI, body mass index; COPD, chronic obstructive pulmonary disease; SIRS, systemic inflammatory response syndrome; ASA, American Society of Anesthesiologists.

**Table 2:** Operative characteristics: CINPT *vs* non‐CINPT patients

	All (*n* = 315)	CINPT (*n* = 77)	Non‐CINPT (*n* = 256)	*P*‐value
Pre‐/postoperative stoma, *n* (%)	184 (58)	71 (92)	113 (48)	<0.01
Elective operation, *n* (%)	220 (70)	49 (64)	171 (72)	0.20
Operative time (min), *n* (%)	166 (88)	220 (97)	149 (78)	<0.01
Contaminated/dirty wound, *n* (%)	166 (53)	43 (56)	123 (52)	0.60

### SSI: bivariate and multivariable analysis

**Figure 3:**
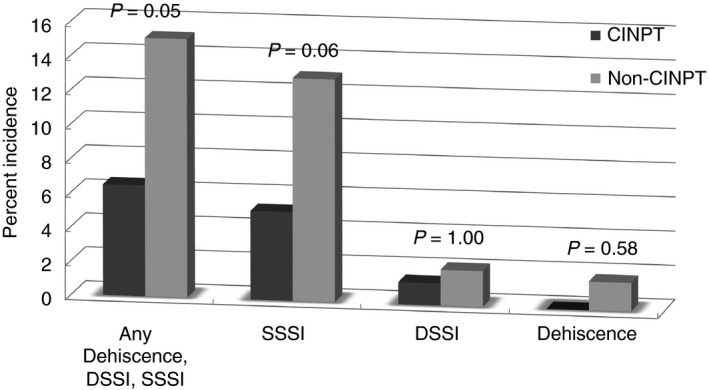
Incidence of SSI: nonmatched CINPT *vs* non‐CINPT (DSSI, deep SSI; SSSI, superficial SSI).

The overall incidence of SSI was 13.0% for the entire cohort (*n* = 41/315). The rate of SSI in the CINPT group was lower (*n* = 5/77) than in the control group (*n* = 36/238) (CINPT *vs* control, 6.5% *vs* 15.1%; *P* = 0.05). The individual components of this composite end‐point are presented in Fig. 3. Time to diagnosis of SSI was longer in the CINPT group than in the control group (CINPT *vs* control, 18.4 *vs* 12.7 days; *P* = 0.05).

Bivariate comparison suggests that patients with SSI had a higher proportion of preoperative dependence on dialysis (*P* = 0.05) and a longer operative time (*P* = 0.06). CINPT usage (*P* = 0.05) was the only other parameter with *P* < 0.10. On multivariable analysis, dialysis dependence (OR 5.90; 95% CI 1.23–28.20) and operative time (OR 1.10; 95% CI 1.02–1.09) were associated with increased SSI whilst CINPT was associated with decreased SSI (OR 0.26; 95% CI 0.10–0.76). The *c*‐statistic for model discrimination was 0.72. The Hosmer–Lemeshow test for model calibration was nonsignificant (*P* = 0.41).

### Secondary outcomes

Mortality, postoperative length of stay and other wound complications were similar between the groups (Table 3). Readmissions were half as frequent in CINPT patients compared with the control group (CINPT *vs* control, 8 *vs* 16%; *P* = 0.09).

**Table 3:** Postoperative outcomes: CINPT *vs* non‐CINPT patients

	All (*n* = 315)	CINPT (*n* = 77)	Non‐CINPT (*n* = 256)	*P*‐value
Organ space SSI, *n* (%)	15 (5)	2 (3)	13 (6)	0.54
Unplanned readmission, *n* (%)	44 (14)	6 (8)	38 (16)	0.09
Length of stay (days), mean (SD)	8.4 (9.0)	8.7 (7.5)	8.3 (9.5)	0.78
Mortality, *n* (%)	4 (1)	0 (0)	4 (2)	0.58

### Van Walraven SSI risk score matched analysis

According to the van Walraven risk score, the matched groups had expected SSI rates of 19.1% for the non‐CINPT group versus 20% for the CINPT group (*P* = 0.39). Preoperative characteristics for the matched cohort are shown in Table 4. The proportion of pelvic cases was similar between groups (CINPT *vs* non‐CINPT, 48% *vs* 44%; *P* = 0.22). The presence of a pre‐ or postoperative stoma was higher in the CINPT group (CINPT *vs* non‐CINPT, 94% *vs* 47%; *P* < 0.01).

**Table 4:** Preoperative and demographic characteristics of matched CINPT *vs* non‐CINPT patients

	All (*n* = 156)	CINPT (*n* = 77)	Non‐CINPT (*n* = 79)	*P*‐value
Age (years), mean (SD)	56.7 (15.1)	56.2 (14.7)	57.3 (15.7)	0.65
Female gender, *n* (%)	70 (45)	31 (40)	39 (49)	0.27
BMI ≥ 30 kg m^–2^, *n* (%)	73 (47)	31 (40)	42 (53)	0.11
Steroid use, *n* (%)	49 (31)	23 (30)	26 (33)	0.73
Diabetes mellitus, *n* (%)	35 (22)	14 (18)	21 (27)	0.25
Race, *n* (%)				
White	128 (82)	66 (86)	62 (79)	0.45
Unknown	12 (8)	6 (8)	6 (8)	
Black	10 (6)	2 (3)	8 (10)	
Asian	4 (3)	2 (3)	2 (3)	
American Indian/Alaskan	2 (1)	1 (1)	1 (1)	
Smoker, *n* (%)	39 (25)	25 (33)	14 (18)	0.04
Dependent functional status, *n* (%)	10 (6)	3 (4)	7 (9)	0.33
History of severe COPD, *n* (%)	8 (5)	4 (5)	4 (5)	1.00
Dialysis, *n* (%)	1 (1)	0 (0)	1 (1)	1.00
Disseminated cancer, *n* (%)	12 (8)	4 (5)	8 (10)	0.37
Open wound, *n* (%)	17 (11)	6 (8)	11 (14)	0.31
Weight loss, *n* (%)	23 (15)	10 (13)	13 (17)	0.65
Bleeding disorder, *n* (%)	9 (6)	3 (4)	6 (8)	0.50
Preoperative transfusion, *n* (%)	5 (3)	2 (3)	3 (4)	1.00
Any preoperative SIRS/sepsis, *n* (%)	26 (17)	12 (16)	14 (18)	0.83
ASA class, *n* (%)				
ASA 1	1 (1)	0 (0)	1 (1)	0.38
ASA 2	41 (26)	24 (31)	17 (22)	
ASA 3	101 (65)	48 (62)	53 (67)	
ASA 4	13 (8)	5 (7)	8 (10)	

BMI, body mass index; COPD, chronic obstructive pulmonary disease; SIRS, systemic inflammatory response syndrome; ASA, American Society of Anesthesiologists.

**Figure 4:**
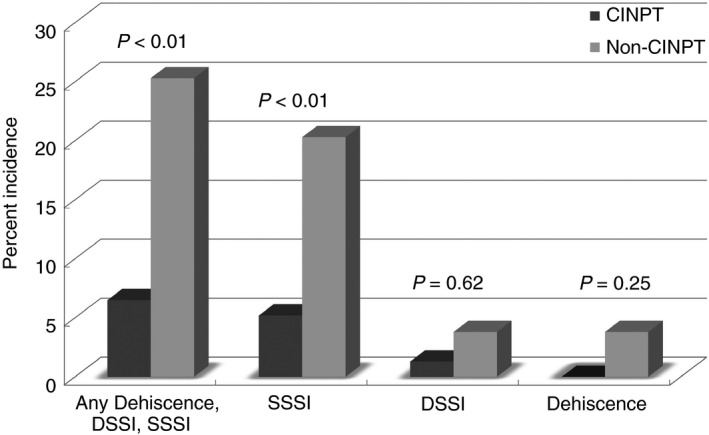
Incidence of SSI: matched CINPT *vs* non‐CINPT (DSSI, deep SSI; SSSI, superficial SSI).

Overall wound complications were 25.3% (*n* = 20/79) in the non‐CINPT group compared with 6.5% (*n* = 5/77) in the CINPT group (*P* < 0.01) (Fig. 4). Factors associated with SSI (*P* < 0.10) on bivariate analysis in the matched cohort included increased operative time (*P* = 0.02) and presence of pre‐ or postoperative stoma (*P* = 0.03). CINPT usage was associated with decreased SSI on bivariate analysis (*P* < 0.01). On multivariable analysis, increased operative time (OR 1.01; 95% CI 1.00–1.01) was associated with increased SSI while CINPT was associated with decreased SSI (OR 0.20; 95% CI 0.06–0.65). Time to SSI diagnosis was longer in the CINPT group (CINPT *vs* non‐CINPT, 18.4 *vs* 11.9 days; *P* = 0.01). Unplanned readmission was increased in the non‐CINPT group (CINPT *vs* non‐CINPT, 8% *vs* 24%; *P* < 0.01).

## Discussion

The results of this study suggest that CINPT in high‐risk patients undergoing open colorectal surgery is associated with decreased incidence of postoperative SSI (CINPT *vs* non‐CINPT, 6.5% *vs* 15.1%; *P* = 0.05), with a greater decrease when matched for SSI risk (CINPT *vs* non‐CINPT, 6.5% *vs* 25.3%; *P* < 0.01). These differences persisted on multivariable analysis of both matched and unmatched cohorts. Placing these results in context, the SSI risk score predicted an SSI risk of 20% for the CINPT group, which is higher than our observed rate of 6.5%, while the predicted rate for the control group was 15%, which was comparable with our observed rate. Such a reduction in SSI in this high‐risk cohort of patients with multiple patient and procedural factors is remarkable. Of note, SSI in the setting of CINPT usage presented almost 1 week later than in patients in the control group (CINPT *vs* control, 18.4 *vs* 12.7 days; *P* = 0.05), which emphasizes the need for ongoing vigilance in postoperative wound surveillance for these patients.

Importantly, we found that the time to diagnosis of SSI was longer in CINPT than control patients by almost 7 days (CINPT *vs* control, 18.4 *vs* 12.7 days; *P* = 0.051). This difference could be attributable to temporary, but incomplete, dead space closure and fluid evacuation during CINPT use. This finding emphasizes the need for awareness and active wound surveillance. There are obvious implications for the timing of the first postoperative visit considering that the mean time to diagnosis of SSI was 18.4 days in the CINPT group.

Our findings are consistent with other studies which have shown a decreased incidence of SSI with the use of CINPT. Hyldig and colleagues performed a meta‐analysis of seven randomized controlled trials including orthopaedics, cardiac, plastic and general surgery cases which compared CINPT with standard wound care. They found that CINPT decreased the incidence of SSI from 8.9% to 4.7% (relative risk 0.54, 95% CI 0.33–0.89) 7. Similarly, Scalise *et al*. reviewed 15 studies across multiple surgical disciplines to show an improvement in SSI rate with CINPT in 80% of the studies reviewed 5. The heterogeneity of these reviews precludes firm conclusions with respect to colorectal surgery patients. Pellino and colleagues reviewed five studies which investigated colorectal surgical patients only, finding a deceased SSI rate associated with CINPT in each study 8. The largest of these, by Bonds *et al*., compared 32 patients having CINPT with 222 patients having standard wound care 22. SSI was identified in 13.8% of CINPT patients and 31% of control patients, with CINPT independently associated with decreased SSI (OR 0.32; 95% CI 0.11–0.96). The largest previously published study, a single‐surgeon experience with CINPT, demonstrated a reduction in SSI from 21% to 3% in 69 high‐risk patients undergoing general and colorectal procedures 23. Conversely, Shen and colleagues performed a randomized controlled trial of CINPT in patients undergoing major intra‐abdominal oncological resection, with no difference demonstrated between the groups (CINPT *vs* control, 12.8% *vs* 12.9%; *P* > 0.99; *n* = 133 *vs* 132) 13. Notwithstanding this result, an international multidisciplinary consensus recommended the use of CINPT for patients at high risk of SSI on the basis of an extensive literature review 24.

It is interesting to speculate on potential cost savings associated with the use of CINPT. It has been estimated that each SSI incurs a cost of approximately $17 000 1, 25. The unit price of CINPT is $495 26. As such a number needed to treat (NNT) of less than 34 patients would generate net savings. As the NNT is simply the inverse of the absolute risk reduction, any absolute risk reduction greater than 2.9% [i.e. (1/34) × 100] would be associated with a net saving. Our data demonstrated an absolute risk reduction of 9% in the unmatched cohort and 19% in the matched cohort, which suggests an opportunity for cost savings. These estimates accord with the findings of Hyldig and colleagues, who demonstrated a NNT of 25 patients (95% CI 17–93) 7. Patient selection for this intervention is highlighted by the meta‐analysis performed by Semsarzadeh *et al*., which demonstrated a 29.4% relative risk reduction yet only a 2.75% absolute risk reduction 27. This suggests that it is important to utilize CINPT in patients at high risk for SSI in order to maximize cost benefits. In a specific cost–utility analysis, Chopra and colleagues arrived at similar conclusions, noting that cost savings are possible for those with a baseline SSI risk exceeding 16.39% 28.

It is important to emphasize that our evaluation of CINPT included only primarily closed wounds. Some advocate leaving contaminated wounds open to heal by secondary intention. Frazee *et al*., in a prospective, randomized trial, demonstrated that CINPT healed wounds faster than open NPT. 29 Similarly, Lewis and colleagues estimated a mean home health cost of $2139 (range $800–$6200) for patients with open wounds, and survey data of wounds managed in the outpatient setting suggested that these often take weeks to heal, all the while incurring expense and diminishing patient satisfaction 34, 35.

We recognize certain limitations to this study. We used a nonrandomized study design with historical controls which might result in treatment bias. Although we attempted to apply CINPT in a uniform, standardized fashion, logistical issues prevented 100% utilization. Though our study groups were similar with respect to measured parameters, there may be differences not captured by the NSQIP database. Further, it is possible that secular trends over the study period may have affected our findings, although it should be emphasized that no new systematic procedural changes in the area of SSI reduction were implemented in our unit during this time. Finally, even assuming a SSI rate of 20% for this high‐risk population, a risk reduction of 50% to a SSI rate of 10% would have required 291 patients per arm for a study with 80% power and an alpha of 0.05. However, given the high utilization of minimally invasive surgery in our division, recruiting nearly 300 open surgery patients was not feasible within a reasonable time frame.

This study aimed to address the potential benefits of CINPT in the context of the available literature. Our study included only high‐risk patients undergoing open colorectal surgical procedures. Such a cohort avoids the drawing of conclusions based upon heterogeneous patient groups. Finally, ours is the first study to utilize NSQIP review in the evaluation of CINPT. The NSQIP provides a standardized assignment of SSI status with uniform 30‐day follow‐up, which will facilitate future comparisons. This is of particular benefit, as it should be noted that there is often significant variability in definitions of SSI and accuracy across data sets 32, 33, 34.

## Conclusions

In this largest study to date of CINPT for patients undergoing colorectal surgery, CINPT was independently associated with a decreased incidence of SSI. Notably, SSI presented later in the setting of CINPT, which emphasizes the need for longer wound surveillance. While we await additional data from the randomized controlled trials already under way to better define the role of CINPT in colorectal surgery patients 35, 36, our data as well as those of others suggest that CINPT provides the potential for cost‐effective quality improvement and a reduction in the incidence of SSI.

## Disclosures

None.
